# *“Everyone Needs a Friend Sometimes”* – Social Predictors of Long-Term Remission In First Episode Psychosis

**DOI:** 10.3389/fpsyg.2016.01491

**Published:** 2016-10-04

**Authors:** Jone Bjornestad, Inge Joa, Tor K. Larsen, Johannes Langeveld, Larry Davidson, Wenche ten Velden Hegelstad, Liss G. Anda, Marius Veseth, Ingrid Melle, Jan O. Johannessen, Kolbjorn Bronnick

**Affiliations:** ^1^Tidlig Oppdagelse og Behandling av Psykoser – Centre for Clinical Research in Psychosis, Stavanger University HospitalStavanger, Norway; ^2^Network for Medical Sciences, University of StavangerStavanger, Norway; ^3^Section of Psychiatry, Department of Clinical Medicine, University of BergenBergen, Norway; ^4^School of Medicine and Institution for Social and Policy Studies, Yale University, New HavenCT, USA; ^5^Faculty of Health and Social Sciences, Bergen University CollegeBergen, Norway; ^6^Norwegian Centre for Mental Disorders Research, University of OsloOslo, Norway

**Keywords:** first-episode psychosis, schizophrenia, social factors, baseline predictors, long-term remission

## Abstract

**Background:** Predictors of long-term symptomatic remission are crucial to the successful tailoring of treatment in first episode psychosis. There is lack of studies distinguishing the predictive effects of different social factors. This prevents a valid evaluating of their independent effects.

**Objectives:** To test specific social baseline predictors of long-term remission. We hypothesized that first, satisfaction with social relations predicts remission; second, that frequency of social interaction predicts remission; and third, that the effect of friend relationship satisfaction and frequency will be greater than that of family relations satisfaction and frequency.

**Material and Methods:** A sample of first episode psychosis (*n* = 186) completed baseline measures of social functioning, as well as clinical assessments. We compared groups of remitted and non-remitted individuals using generalized estimating equations analyses.

**Results:** Frequency of social interaction with friends was a significant positive predictor of remission over a two-year period. Neither global perceived social satisfaction nor frequency of family interaction showed significant effects.

**Conclusions:** The study findings are of particular clinical importance since frequency of friendship interaction is a possibly malleable factor. Frequency of interaction could be affected through behavioral modification and therapy already from an early stage in the course, and thus increase remission rates.

## Introduction

Social predictors of long-term remission are decisive to tailor treatment in first-episode psychosis (FEP). Patients tend to have smaller friendship and family networks, and less social interaction, compared to the general population (Fraser et al., 2006; Palumbo et al., 2015). Throughout the course of illness, they may find it difficult to develop and maintain social relationships, and their social networks, particularly friendships, often diminish in size (Gayer-Anderson and Morgan, 2013). This negative development has been shown to pre-date the onset of psychosis (Gayer-Anderson and Morgan, 2013), and to be associated with increased hospitalization rates and worse outcome (Albert et al., 1998; Fraser et al., 2006). This relative deficit in social relationships, however, represents a potential target for intervention, as inclusive and supportive friendship and family networks are associated with better outcomes and more efficient use of health services (Evert et al., 2003; Pinto, 2006; Marino et al., 2015). Further, robust social networks are associated with reduction in subjective loneliness (Davidson et al., 2008; Hawkley and Cacioppo, 2010), decrease in perceived social stigma (Watson et al., 2007), as well as an increase in self-care functioning (Evert et al., 2003). Studies show that even if family networks are robust some findings indicate friendships to be more strongly associated with symptom reduction, improved social functioning, and independence (Erickson et al., 1989; Morgan et al., 2008; Reininghaus et al., 2008). Both practical support and emotional friendships buffer harmful impacts of stress exposure, and increase overall well-being (Davidson et al., 2004; Thoits, 2011). A review on peer support among individuals with severe mental illness shows similar results (Davidson et al., 1999).

Several reviews have characterized the literature in this field as heterogeneous (Buchanan, 1995; Tew et al., 2011; Thoits, 2011; Gayer-Anderson and Morgan, 2013; Palumbo et al., 2015). A main critique concerns a study approach merging variables such as family relations, friendships, frequency of interactions, and satisfaction with interactions into one, or few, global categories. This prevents a valid evaluation of their independent effects on outcome. In addition, studies are often based on heterogeneous samples consisting of both chronic and first episode psychosis, limiting generalizability of study findings. Ultimately, these knowledge gaps may impede helpful intervention choices, and research to identify specific social factors beneficial to outcome seems called for.

This study was designed to investigate effects of friendships relative to family relations, as well as the effects of relational frequency relative to relational satisfaction, in an epidemiological FEP sample. To do so, we developed three hypotheses of baseline predictors of long-term remission.

### Hypotheses

We hypothesized that first, satisfaction with social relations predicts remission; second, that frequency of social interaction predicts remission; and third, that the effect of friend relationship satisfaction and frequency will be greater than that of family relations satisfaction and frequency.

## Materials and Methods

### Sample

The sample was recruited from the on-going TIPS-2 study (early Treatment and Intervention in Psychosis), a naturalistic follow-along FEP study in south-Rogaland, Norway, including individuals with FEP from January 2002, until August 2013. Detailed descriptions of the inclusion criteria and methods have been published elsewhere (Joa et al., 2008; Stain et al., 2013). TIPS-2 was approved by the Regional Committee for Medical Research Ethics Health Region West, Norway (015.03). Written informed consent was obtained for all participants.

Individuals who were included in the study met the following criteria: living in the catchment area (Rogaland county); age 15–65 years; meeting the DSM-IV criteria for a first episode of schizophrenia, schizophreniform psychosis, schizoaffective psychosis, delusional disorder, brief psychosis, affective disorder with mood incongruent delusions, or psychosis not otherwise specified, and also from August 1, 2008 substance induced psychosis; being actively psychotic as measured by the Positive and Negative Syndrome Scale (PANSS) (Kay et al., 1987); not previously receiving adequate treatment of psychosis; no neurological or endocrine disorders related to the psychosis; understands and speaks one of the Scandinavian languages; an IQ over 70; and being able and willing to sign an informed consent. The patients agreed to baseline assessment, and follow-up after 3 months, and 1, 2, and 5 years.

**FIGURE 1:**
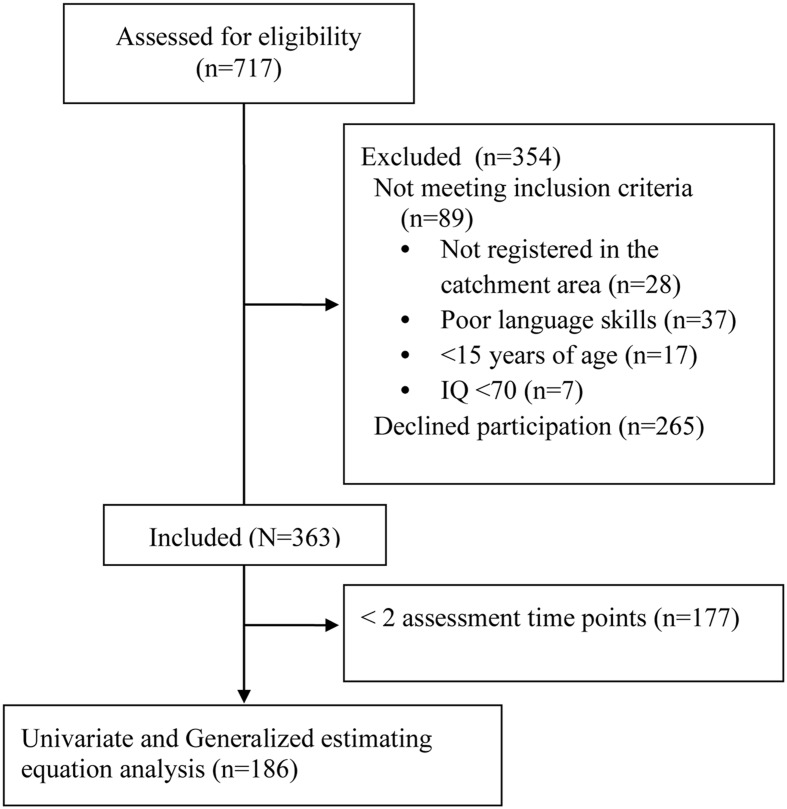
**Flowchart – Participant participation**.

See **Figure 1** for participant participation. In this sub-study for the purposes of our statistical analyses, we only included individuals with a minimum of one measurement of one- and/or two-year remission and a complete set of data for all predictor and covariate variables, leaving a total of 186 individuals. The 177 individuals that did not take part in the analysis did not significantly differ from those included in analyses on demographic or clinical characteristics [Age, Gender, PANSS scales (positive, negative, depressive, excitative, disorganized), GAF symptom, GAF function and Duration of untreated psychosis (DUP)]. Attrition is hence seemingly random, and the resulting sample can be assumed to be representative.

### Baseline Measures

The Structured Clinical Interview for DSM-IV Axis I Disorders (SCID-I) (First et al., 1995) was used for diagnostic purposes. PANSS was used to assess severity of positive and negative symptoms of psychosis. In general, PANSS has been found to have good reliability and validity. Interrater reliability agreement for individual items ranges from 0.31 to 0.93; positive symptom scale, 0.72; negative symptom scale, 0.80; and the general psychopathology scale, 0.56. Global internal consistency for the positive symptom symptom scale is 0.62; negative symptom scale, 0.92; and the general psychopathology scale 0.55. (Kay et al., 1988; Peralta and Cuesta, 1994).

To determine group differences between remitted and non-remitted participants with regard to antipsychotic treatment, and psychotherapy at one-year follow-up, we defined the following durations; weeks from inclusion to starting antipsychotic treatment and psychotherapy and weeks duration of antipsychotic treatment and psychotherapy. These data were not available for the second year of treatment.

Global functioning was measured by the Global Assessment of Functioning Scale (APA, 1994). Scores were split into symptom (GAFs) and function (GAFf) subscales (Melle et al., 2004). The Clinicians Rating Scale (Drake et al., 1990) was used to measure alcohol and other substance abuse. To measure substance abuse/no substance abuse this variable was transformed to a binary variable with abuse defined as a score of >2. DUP was estimated based on information from the PANSS and SCID-I interviews as the time from onset of psychosis until the start of adequate treatment (Larsen et al., 2001). Onset of psychosis was considered to be the first appearance of positive psychotic symptoms, corresponding to a PANSS score of four or more on at least one of the following PANSS items; P1 (delusions), P3 (hallucinations), P5 (grandiosity), P6 (suspiciousness), and A9 (unusual thought content), for at least 7 days.

The brief version of Lehman’s Quality of Life Interview (L-QoLI) (Lehman, 1996) was used to measure objective (e.g., frequency of contact with family and friends) and subjective (e.g., satisfaction with social relations) social functioning, and to differentiate between family and friends. We used five L-QoLI subscales: Satisfaction with family relations, social relations, and with daily activities were subjective measures; and frequency of family and of social contacts (friends) were objective measures. Subjective measures were rated on a 7-point scale, ranging from 1 (terrible) to 7 (delighted) (Lehman et al., 1995). The psychometric properties of the L-QoLI have been assessed. Internal consistency ranges from 0.79 to 0.88 (median 0.85) for the life satisfaction scales, and from 0.44 to 0.82 (median 0.68) for the objective quality of life scales. Test-retest reliabilities (1 week) range from 0.41 to 0.95 (median 0.72) for life satisfaction; and 0.29 to 0.98 (median 0.65) for objective scale (Lehman, 1996).

### Procedure

Trained personnel conducted baseline assessments within a week of contact. Raters were trained by rating pre-prepared case notes, videotaped interviews, vignettes *and follow-along in-vivo observation of clinical interviews* before entering the study assessment team. Good inter-rater reliability was achieved on major parameters in the research group in 2008 (Joa et al., 2008) and 2012. Reliability of measurements for DUP was 0.8 (ICC), and for diagnostic categories; *K* = 0.9 (Weibell et al., 2013). All participants included at baseline were set up for three-month, one-, two-, and five-year follow up evaluations. Baseline and follow up assessments (commonly lasting 2–3 h) mainly took place at Stavanger University Hospital, but sometimes at participants homes or other locations assigned by the participants.

### Predictor Variables

Predictor variables were baseline social and family relations measures from L-QoLI: (a) Frequency of interaction with friends (Friends frequency), (b) frequency of interaction with family (Family frequency), (c) satisfaction with friend relationships (Friends satisfaction), and (d) satisfaction with family relationships (Family satisfaction). These variables enabled us to independently test the contributions of family and friends. In order to investigate total frequency and satisfaction of social interaction, we also computed mean scores for each dimension, based on the sum of family and friends scores (Social satisfaction total and Social frequency total).

### Outcome Measures: Defining Remission at Last Available Observation

Remission was defined in accordance with The Remission Working Group standardized symptom remission criteria (Andreasen et al., 2005): no score of four or higher for the past 6 months on any of the following PANSS items: P1 (delusions), P2 (disorganized thought), P3 (hallucinatory behavior), N1 (affective flattening), N4 (passive social withdrawal), N6 (lack of spontaneity), G5 (bizarre posture), or G9 (unusual thought content). Individuals were categorized as non-remitted if they reported any relapse, defined as deterioration of symptoms scored >3 on the relevant PANSS scales, during the previous 6 months. Remission status at the last available observation was based on one- and two-year follow up evaluations.

### Statistical Analysis

Analyses were carried out using SPSS v. 22 (IBM Corp. Released, 2014). For the univariate analysis of baseline variables, comparing remitted to non-remitted subjects, the remitted group was defined according to remission status at the last available observation at 12 or 24 months. Baseline and follow-up between-group differences were estimated employing Pearson χ^2^ tests for categorical variables, and unpaired two-tailed *t-*tests for continuous variables. Non-parametric statistics (Mann–Whitney *U*-test) were applied for comparison of non-normally distributed data (Kolmogorov–Smirnov test). The DUP variable was log transformed due to severely skewed distribution. All tests were two-tailed, and Bonferroni corrections were carried out when appropriate. The choice of correction factor was made in each case based on the number of tests pertaining to each hypothesis, or number of variables in each category of parameters (see **Table 1**).

**Table 1:** Demographic, baseline clinical characteristics, predictor variables.

		Symptomatic Remission		
			
Characteristic	Total	No	Yes	P (E.S)
Remission last available obs. % (*n*)	186	58.6 (109)	41.4 (77)	
**Demographics (α = 0.025)**				
Age of onset (SD)	26.92 (10.76)	26.21 (10.38)	27.94 (11.26)	0.283 (0.160)
Female % (*n*)	45.2 (84)	59.5 (50)	40.5 (34)	
Male % (*n*)	54.8 (102)	57.8 (59)	42.2 (43)	0.979 (0.004)
**Clinical status (α = 0.005)**				
GAF Symptom mean (SD)	31.82 (7.12)	32.06 (6.85)	31.50 (7.11)	0.554 (0.079)
GAF Function mean (SD)	41.10 (10.84)	40.28 (10.14)	42.23 (11.69)	0.175 (0.184)
**PANSS mean item score (SD)**				
Negative	2.15 (1.07)	2.18 (1.00)	2.10 (1.16)	0.602 (0.078)
Disorganized	2.02 (1.05)	1.93 (0.91)	2.09 (1.14)	0.158 (0.213)
Depressive	3.26 (1.04)	3.31 (1.07)	3.20 (1.01)	0.453 (0.112)
Positive	3.16 (0.82)	3.19 (0.86)	3.11 (0.75)	0.523 (0.095)
Excitative	1.58 (0.73)	1.53 (0.67)	1.65 (0.80)	0.288 (0.159)
Total	64.69 (14.25)	64.39 (11.62)	65.13 (17.39)	0.727 (0.053)
^a^Substance abuse % (*n*)	24.2 (45)	60.00 (27)	40.00 (18)	0.630 (0.064)
^b^DUP weeks median (Range, SD)	20.00 (0–2080, 221,05)	30.00 (0–2080, 264.17)	13.50 (0–572, 135.17)	0.010 (0.253)
^c^Core schizophrenia % (*n*)	32.30 (60)	63.3	36.7	0.143 (0.193)
**Predictor variables (α = 0.008)**				
**^d^ Social satisfaction (SD)**				
Family	4.78 (1.36)	4.61 (1.47)	5.02 (1.15)	0.040 (0.309)
Friends	4.617 (1.25)	4.45 (1.32)	4.85 (1.11)	0.029 (0.328)
Social satisfaction total	4.69 (1.22)	4.52 (1.12)	4.92 (0.83)	**0.008**(0.404)
**^e^Social frequency (SD)**				
Family	4.12 (0.84)	4.05 (0.92)	4.21 (0.71)	0.178 (0.203)
Friends	3.41 (1.05)	3.23 (1.13)	3.67 (0.88)	**0.005** (0.428)
Social frequency total	3.69 (0.77)	3.56 (0.83)	3.88 (0.62)	**0.004** (0.441)


*^a^Transformed to binary variables: Abuse defined as score of >2 measured by the Clinicians Rating Scale.*

*^b^DUP: *p*-value and effect size is based on log-transformed DUP.*

*^c^Schizophrenia spectrum disorders: Schizophrenia, Schizophreniform, and Schizoaffective disorder.*

*^d/e^Variables from Lehman quality of life interview. ^∗^Bonferroni adjusted α levels for each section of the table. E.S: Effect size (Cohen’s D).*

*Bold values indicate statistically significant values.*

Generalized Estimating Equations (GEE) (Zeger and Liang, 1986; Diggle and Kenward, 1994), set up as a binary logistic model, with a robust estimator and an unstructured covariance matrix, were used to test predictors of remission at one- and/or two-year follow-up. GEE uses regression equations to estimate values of missing assessment data, and allows for repeated measurements in the model. This compensates for weaknesses in using status at the last observation as an outcome measure, as remission at all time points can be analyzed and missing data handled in the model. Model covariates were age, gender, baseline symptoms (PANSS positive, negative, depressive, excitative, and disorganized), DUPlog, time and substance abuse. Predictor variables and covariates were entered simultaneously in the GEE model as main effects, alongside the interaction terms of time with the hypothesis derived predictors. We conceptualized two dimensions of social functioning: Frequency vs. satisfaction, and friends vs. family. This gave rise to four combinations of social interactions for use in the statistical models, and yielded the opportunity to investigate systematically the relative importance of frequency of, versus satisfaction with, family vs. friend interactions.

#### Satisfaction versus Frequency

Model 1: In order to pin-point the specific effects of the dimensions satisfaction versus frequency, we collapsed friends and family into one dimension, resulting in the variables: Social Frequency and Social Satisfaction. These were entered as predictors.

#### Family versus Friend Contacts

Model 2: In the second model, the relative importance of satisfaction with friends versus satisfaction with family contacts was investigated. Hence, the predictors Friends Satisfaction and Family Satisfaction were entered into the model.

Model 3: A third model was fitted to estimate the relative importance of frequency of interactions with friends versus family. Hence, the predictors Friends Frequency and Family Frequency were entered.

Model 4: From each of the fitted models, the significant predictors were included in a final analysis, excluding the other predictor variables, but still including the cofounder variables.

## Results

Baseline characteristics and univariate baseline analyses comparing the remitted and non-remitted groups are displayed in **Table 1** (*n* = 186). A total of 34.2 percent (77/225 individuals) met the standardized symptom remission criteria (Andreasen et al., 2005) at 1 year, and 47.3 percent (79/167 individuals) were remitted at 2 years.

Remitted individuals scored significantly higher than non-remitted individuals on the following predictor variables (according to the Bonferroni adjusted α limit): (1) Friends and family satisfaction mean score, (2) Friends frequency, and (3) Friends and family frequency mean score. For the first year of treatment, we found no significant between-group differences for weeks from inclusion to starting antipsychotic treatment (*p* = 0.*342*; OR = 0.140) and psychotherapy (*p* = 0.*343*; OR = 0.139), or for weeks duration of antipsychotic treatment (*p* = 0.*466*; OR = 0.106) and psychotherapy (*p* = 0.*324*; OR = 0.142).

### Predictors of Remission

**Table 2** shows the results of the adjusted GEE model used to measure predictors of remission over the two-year follow-up (*n* = 186). Analysis revealed no significant interaction effects between any predictor variable and time. We therefore removed all interaction terms from the model.

**Table 2:** Predictor effect derived from generalized estimating equations analyses on remission status over the 2 year follow up.

Characteristic	Test of model effects			Parameter estimates		

***n* = 186**	**Wald χ^2^**	**df**	***p***	**Odds ratio**	**Confidence interval (95%)**	
Gender	0.001	1	0.978	1.009	0.528	1.929
Substance abuse	0.468	1	0.494	1.296	0.616	2.727
PANSS negative	0.271	1	0.603	1.079	0.810	1.439
PANSS disorganized	0.409	1	0.523	0.899	0.647	1.247
PANSS depressive	0.378	1	0.539	1.106	0.802	1.525
PANSS positive	0.788	1	0.375	1.198	0.804	1.786
PANSS exititative	0.173	1	0.678	0.911	0.585	1.416
DUP	7.444	1	**0.006**	1.727	1.166	2.557
Age	5.656	1	**0.017**	0.960	0.928	0.993
Social frequency friends	10.314	1	**0.001**	0.599	0.438	0.819
**^∗^*Time***					
1 year	6.977	1	**0.008**	1.545	1.119	2.133


*^∗^Time reference category = 2 years follow up.*

*Bold values indicate statistically significant values.*

Regarding hypothesis one, *Social satisfaction total* did not predict remission. Regarding hypothesis two, *Social frequency total* did predict remission. Regarding hypothesis three, when separately including friends and family satisfaction predictor variables in the GEE analysis, results were non-significant for all predictors. The next GEE model included the predictor variables *Social Frequency Family* and *Social Frequency Friends* separately. Only *Social Frequency Friends* showed a significant effect as an independent predictor. Thus, we excluded the predictor variable *Social Frequency Family* from the model (the final model outlined in **Table 2**).

## Discussion

### Social Frequency Beneficial Regardless of Perceived Social Satisfaction

Describing someone as a friend commonly implies some degree of relational satisfaction. Therefore it may initially seem paradoxical that frequency of friendship interaction should outperform social satisfaction as a predictor of remission. One possible interpretation is that although some participants perceived their social interactions as unsatisfactory, these interactions nevertheless have beneficial effects which participants were unable to fully appreciate or detect. In line with this perspective, previous research in psychosis has found that high baseline symptom load, including a combination of social withdrawal and anhedonia (Kirkpatrick et al., 2006; Haro et al., 2015) with perceptual and attribution disturbances (Frith, 2014; Strassnig et al., 2015), is related to participants reporting a negatively biased and distorted view of social reality (Malla et al., 2004). This finding is also consistent with research on highly introverted individuals, similarly demonstrating a significant association between social frequency, relationships, and well-being, which appears to contradict their subjective assessment that social interaction has a limited impact on their well-being (Hotard et al., 1989). Thus, subjective reports alone may not have illuminated the full positive effects of social relationships in our sample.

It is however, plausible that frequent social contact increase subjective relational satisfaction by an *mere exposure effect* (Zajonc, 1968), which in turn may explain in part why social frequency robustly predicts remission. The exposure effect posits that individuals generally tend to develop preferences for objects and individuals as a consequence of familiarity. However, research shows that this effect requires and is reinforced by consistency in social relations, and conversely, is reduced if scattered among many (Zajonc, 1968; Bornstein, 1989). Hence, increased social frequency, if consistently maintained, may in itself increase subjective satisfaction with social relationships. This interpretation emphasizes the potential importance of targeting frequency of interaction in early psychosocial interventions.

### Clinical Implications

Our findings opens the possibility of investigating whether remission rates might be improved through systematic treatment efforts aimed at increasing early social frequency. In related research on severe mental illnesses associations has been found between supported socialization, increased social functioning, self-esteem and chances for achieving a positive long-term outcome (Davidson et al., 2004; Tew et al., 2011), indicating that social frequency may be a modifiable treatment factor. Further, as social frequency is readily quantifiable (Lehman, 1996), this factor is well suited for objective tracking of progress.

Given the risk of deterioration of patients’ social network (Gayer-Anderson and Morgan, 2013) and the general benefits of early intervention in FEP (Marshall et al., 2005; Jääskeläinen et al., 2012), study findings advocates a strong argument that this type of intervention should be applied as early as possible in the course of illness. Although interventions generally ought to be tailored to the needs and wishes of the individual (Davidson et al., 2004; Fraser et al., 2006; Austin et al., 2015), our findings indicate that even individuals with a negative assessment of social quality, and individuals with an inclination for social withdrawal and isolation, should nonetheless be encouraged to participate in social interaction interventions. Thus, study findings imply that professionals perhaps should deemphasize the subjective assessments of relational qualities in the early customization of treatment and evaluation of treatment efficacy in the initial phase of the course.

### Limitations

There are some limitations with the study: (1) This study was performed in an early intervention area, meaning that many participants received an extensive standardized treatment package. This may decrease generalizability to populations not covered by this type of health care. (2) Although adjusted for several research based covariates, premorbid adjustment level was not adjusted for in the main analysis (GEE) due to our assessment that these variables possibly showed multicollinearity with the predictor variables. (3) Also, in this study social satisfaction data were collected at baseline, and equivalent investigations at a later stage might have revealed different ratings of essentially similar social experiences. (4) Finally, there is a possibility that patients who experienced remission also experienced change of social interaction, which may imply that changes in social interaction may be related to remission.

Future research might investigate in particular whether social frequency also affects functional remission in psychosis. Also, in-depth investigations of friendship relations might help reveal any unique relational qualities, which make them more particularly important to remission.

## Author Contributions

All authors have made substantial contributions to all phases of the paper. JJ, TL, IJ, and WtV were involved in funding of TIPS-2. JB and KB conceptualized/performed the current analyses and wrote the first draft. All authors were involved in study design, provided scientific oversight throughout the project, detailed comments to the paper across several drafts and edited the paper.

## Conflict of Interest Statement

The authors declare that the research was conducted in the absence of any commercial or financial relationships that could be construed as a potential conflict of interest.
